# Healthy and unhealthy eating after a behaviour change intervention in primary care

**DOI:** 10.1017/S1463423622000147

**Published:** 2022-03-31

**Authors:** Gro Beate Samdal, Ole Johan Furset, Marte Blom Nysæther, Eirik Abildsnes, Thomas Mildestvedt, Eivind Meland

**Affiliations:** 1 VID Specialized University, Faculty of Health, Bergen, Norway; 2 Department of Global Public Health and Primary Care, University of Bergen, Norway; 3 Department of Psychosocial Health, University of Agder, Kristiansand Municipality, Norway

**Keywords:** behaviour change, healthy diet, non-communicable diseases, physical activity, primary health care, self-rated health

## Abstract

**Background:**

To prevent and reduce non-communicable diseases, the Norwegian Directorate of Health encourages Healthy Life Centres (HLCs) in all municipalities.

**Aims:**

This study investigates whether the behaviour change interventions at HLCs positively affected participants’ diet and to evaluate predictors for healthy and unhealthy eating. Our data are part of the Norwegian Healthy Life Centre Study, a 6-month, pragmatic randomised controlled trial (RCT).

**Methods:**

Totally, 118 participants ≥18 years old were randomised to an intervention group (*n* 57), or a waiting list (control group) (*n* 61). Eighty-six participants met at the 6 months follow-up visit. We merged the participants to one cohort for predictor analyses, using linear regressions.

**Findings:**

The RCT of the HLCs’ interventions had no effect on healthy and unhealthy eating 6 months after baseline compared with controls. A short, additional healthy eating education programme produced a modest, statistically significant improvement in healthy eating compared with controls. This did not, however, reduce unhealthy eating. Higher income predicted unhealthier eating over time. Increasing body mass index and impaired physical functioning also led to an increase in unhealthy eating. Healthy eating at 6 months was predicted by self-rated health (SRH), vitality and life satisfaction, and hampered by musculo-skeletal challenges and impaired self-esteem (SE). SRH impacted improvement in healthy eating during the 6 months. The effect of interventions on healthier eating may be improved by an emphasis on developing positive self-concepts like better SRH, vitality, life satisfaction, and SE.

## Introduction

Norway supports the World Health Organization’s (WHO) global action plan for prevention and control of non-communicable diseases (NCDs) (2013). In 2012, a Public Health Report followed by a Public Health Act, called for a *Health in all policies* approach. The report increased local government responsibility for public health care (Folkehelseloven, 2011). Compared with WHO’s global action plan against NCDs, the new Norwegian NCD strategy placed a strong emphasis on individualised preventive measures towards physical activity (PA), healthy diet, tobacco cessation, and reduced alcohol consumption (2013). The government recommends that municipalities develop a new primary health care service for people at risk of NCDs, or for those who have had disease and need support in order to change their health behaviour (Veileder for kommunale frisklivssentraler, 2011). Through economic incentives from the government over several years, the service has spread into routine practice. In 2016, more than 57% of municipalities had a Healthy Life Centre (HLC) service, an increase of 118% from 2011 to 2014, and 27 000 persons attended the interventions (Ekornrud and Thonstad, 2016). Public health insurance covers all costs for users of HLCs. However, some HLCs do request a small fee (ca. €50).

The PA interventions at the HLCs are the Norwegian model of what other countries have called green prescription (New Zealand), exercise referral scheme (ERS) or PA referral scheme (United Kingdom), or PA on prescription (Sweden). The HLCs aim to support individuals with or at risk for NCDs who need help to change their health behaviour. However, in addition to PA, the HLCs also support change in healthy eating, tobacco cessation, or reduced alcohol consumption.

Internationally, there has been considerable uncertainty as to the effectiveness of prescriptions/referrals to increasing PA, and not enough evidence to indicate whether this type of intervention is more effective than other primary care interventions, for example advice from primary care doctors or nurses (Pavey *et al.*, 2011; Orrow *et al.*, 2013; Campbell *et al*., 2015). Two meta-analyses on the effect of diet interventions evidence a small effect on healthy eating at short term, but this effect was reduced or vanished completely at later time points (Desroches *et al*., 2013; Samdal *et al*., 2017). Concerns have been raised about the widespread rollout of such programmes due to limited evidence (Pavey *et al.*, 2012; Denison *et al*., 2014). Despite the critics, prescriptions/referrals have become increasingly popular.

The study of the HLCs is a relatively young research field with few peer-reviewed studies. The Norwegian Healthy Life Centre Study was a 6-month, pragmatic, randomised controlled trial (RCT) with a longitudinal cohort study (24 months from baseline) (Abildsnes *et al*., 2017). An RCT found no effect on change in PA 6 months after baseline, compared with the control group, but the subgroup least physically active at baseline benefitted significantly from the interventions, as compared with more active participants (Samdal *et al*., 2018a). The interventions did not affect body mass on average, but promoted weight loss among the leaner participants, compared with those with a higher body mass index (BMI) in the intervention group (Samdal, 2021). This study also revealed that several negative self-concepts, for example body shape concern, impaired weight related self-esteem (SE), and controlled motivation for change predicted an impairment of participants’ body attitude after 6 months.

A frequent reason for participants’ attendance at the HLCs was to learn about healthy eating (Samdal *et al*., 2018b). Twenty-five per cent of adult Norwegians are obese and consume large amounts of sugar and salt, red meat and saturated fat in their diet, and have a low intake of fruit and vegetables (2019). Healthy eating is emphasised as one of the main tasks for HLC interventions, but not all HLCs offer structured counselling on the topic. Former HLC studies have mainly focused on PA and quality of life (Samdal *et al.*, 2018a; Blom *et al*., 2020). The present study is the first to evaluate the effect on participants’ healthy and unhealthy eating 6 months after baseline and examined if socio-demographic and other characteristics could explain the effects. We performed both temporal causal and residual change analyses.

## Methods

The study is reported in accordance with the Consolidated Standard of Reporting Trials statement and the Template for Intervention Description and Replication. The protocol is available online at ClinicalTrials.gov (ID: NCT02247219). Additional information about design, recruitment, the interventions, and data collection has been presented in previous studies (Abildsnes *et al*., 2017; Samdal *et al*., 2018a; Samdal *et al.*, 2018b).

### Setting and recruitment

A convenient sample of eight municipalities accepted the invitation. These are located on Norway’s South and Western coasts and represent 630 000 inhabitants from rural and urban areas. The HLCs invited 351 participants to the study from June 2014 to September 2015, and 118 were included. Inclusion criteria were ≥18 years old, no severe mental illness or learning disabilities, and able to participate in a group-based intervention held in the Norwegian language.

### Randomisation

**Figure 1.**
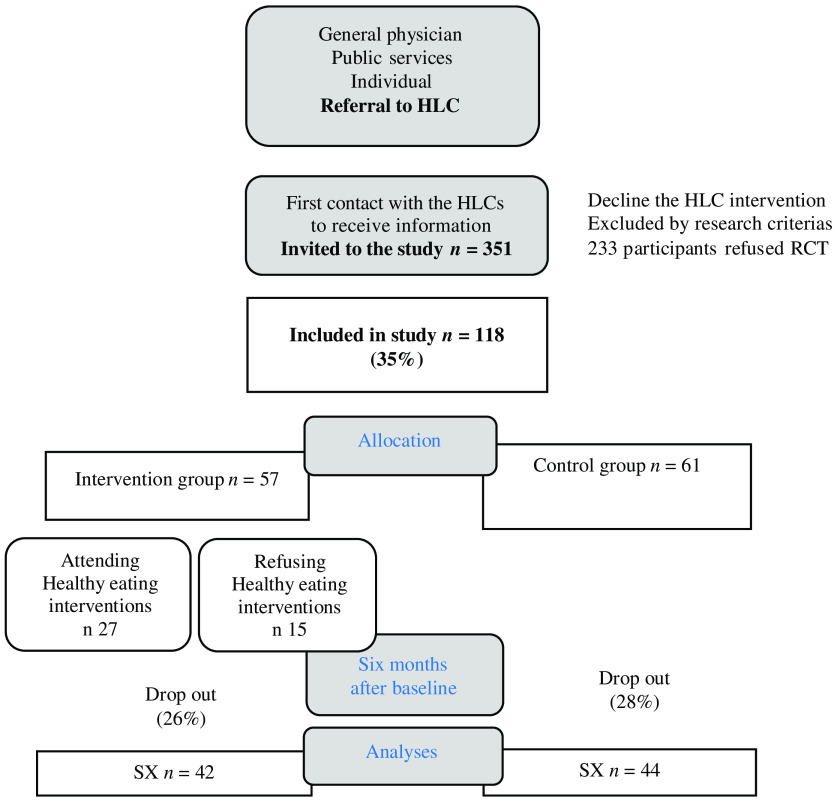
Flow chart of referral, uptake, dropout and attendance. Abbreviations and symbols: HLC = healthy life centre; RCT = randomized controlled trial; SX = SurveyXact online survey.

Randomisation was performed by a project coordinator at the University of Bergen, who drew a card from numbered, sealed, opaque envelopes. Participants on the waiting list (*n* 61, control group) received the interventions after 6 months (Figure 1). All participants in the intervention group (*n* 57) attended group-based PA. They were also offered a healthy eating intervention, but not all of them accepted and we divided the intervention group into two subgroups: Ia) those who accepted and Ib) those who abstained from the healthy eating intervention.

### Data collection

This study presents data collected at baseline and 6 months after baseline (RCT). Questionnaires were administered using an online survey management system (SurveyXact TM; Rambøll Management Consulting, Norway) and completed at the HLCs.

### Interventions

**Figure 2.**
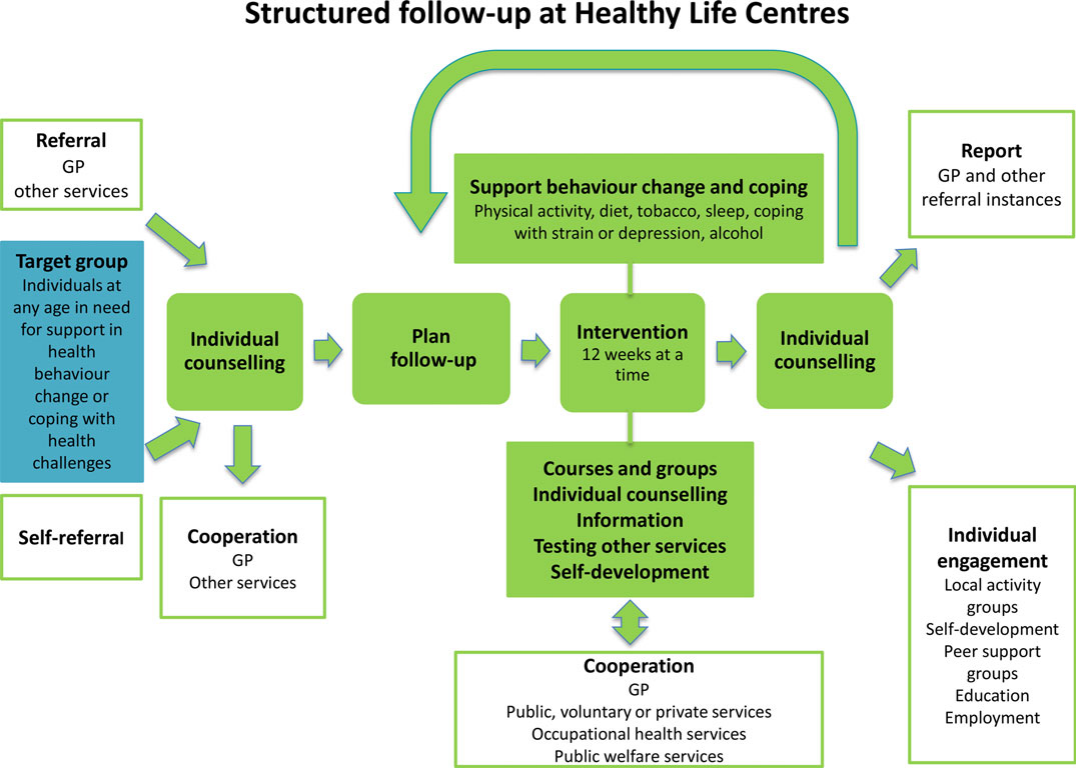
The Norwegian Directorate of Health’s model for the Healthy Life Centres

The HLC model consists of (1) referral by a general practitioner (GP), other public personnel or self-referral; (2) individual counselling at entry and exit; (3) support through behavioural change interventions promoting PA, healthy diet or smoking cessation; and (4) a 12-week intervention period (prescription period) (Veileder for kommunale frisklivssentraler, 2011) (Figure 2). The HLC model is not based on a theory of health behaviour or a theoretical framework for health behaviour change. The Directorate’s basic recommendation does, however, mention several cognitive theories and presents the transtheoretical model of change (TTM) (Prochaska and DiClemente, 1982) as a way of understanding the process of changing, in addition to motivational interviewing (MI) (Miller and Rollnick, 2012) as a general counselling approach (2016 IS: 1896). The individual MI counselling (30–60 min) may also include several techniques from cognitive behavioural therapy. The Directorate of Health recommends that counsellors start the counselling sessions at entry by acknowledging the participant’s perspective of health, offering information about health consequences, and presenting the intervention support. Based on readiness to change, and a discussion about personal barriers/facilitators for change, the participant and counsellor agree on a goal for behaviour change. Some HLCs confirm behaviour goals in a written action plan. In addition, the Directorate of Health encourages the use of free self-help material, for example recipes and cookbooks, and web-based applications for self-monitoring of diet.

Over 50% of the employees at the HLCs are physiotherapists, but apart from this, counsellors’ professional backgrounds vary, for example nurses trained in public health or trained lifestyle counsellors (Abildsnes *et al.*, 2017). The interventions are based on national health recommendations (2002; 2014). The content (what) and organisation (how) of the interventions vary according to resources and competence in the municipalities. A physical therapist (or other professional) facilitates social support for PA through group-based interventions (Nordic walking, light strength conditioning, stretching, games), which often take place outdoors regardless of weather. Participants must attend at least two PA group sessions a week, which often take place outdoors regardless of weather. The Directorate of Health has also developed a healthy eating educational course ready for local implementation (2016 IS: 1896). The participants are offered a group-based educational course by a nutrition expert, which includes recommendations for a healthy diet, meal regularity, composition and portion size, and how to interpret food product labelling in a 2-h weekly session over 4 to 5 weeks. HLCs with access to kitchen facilities could also demonstrate how to make healthy food. The educational course was a voluntary add-on to the PA group sessions.

After 12 weeks, a second individual counselling session provides the opportunity to review behaviour goals. Improvements in outcome of behaviour, for example fitness, well-being, diet, or weight loss are evaluated. The counsellors offer feedback and praise efforts and results in order to build self-efficacy for change. The majority of HLC prescriptions last more than 12 weeks (Denison *et al*., 2014). If there is a need for further or another type of intervention, the participant may extend the prescription period several times, up to 1 year.

### Primary outcome: healthy and unhealthy eating

The survey measured habitual diet, beverage consumption, and eating behaviour. Questions measuring meal composition and beverages have been previously validated in Norwegian Health Surveys (Lorentzen *et al*., 2007). Consumption of healthy and unhealthy eating was measured by questions such as “How often do you eat: (a) fruit and berries/vegetables (Cronbach’s alpha of 0.73) and (b) cakes, biscuits, buns, and fast food (Cronbach’s alpha of 0.78). The participants could answer “never”, “seldom”, or report frequency per day, or per week. We identified these two constructs by an explorative factor analysis with Oblimin rotation among 12 items belonging to the Mediterranean Diet Score (Martínez-González *et al.*, 2012). The two factors, each with two items, exhibited factor loadings >0.8, and with negligible cross loadings. Both constructs were strongly skewed (skewness >3.0). Therefore, we log-transformed the constructs and reported standardised beta coefficients (b) in all analyses. The skewness of both log-transformed outcomes was <1.0.

### Adjusting variables

Gender, age, and randomisation allocation were used as adjusting variables. In the residual change analyses, outcome measures at baseline were entered in the regressions.

### Predicting variables

At inclusion and after 6 months, the counsellors measured participants’ weight and height to ascertain the BMI. In the survey, socio-economic status (SES) was defined by level of education and income. Education level (a five-item scale) was compressed into three categories in line with the Norwegian education system: (1) low: upper-secondary school and below; (2) middle: upper-secondary school with general studies; and (3) high: university college and/or university. Gross family income was defined by a seven-item scale (NOK < 201 000 to > 850 000). In Table 1, we have presented income in two categories < and ≥ 400 000 NOK.

**Table 1.** Descriptive baseline statistics of 86 completers in the Norwegian healthy life centre study (RCT) recruited from June 2014 to September 2015 and according to intervention and control group

Variable (scale) category	Total *n* = 86 (100%)		Intervention *n* = 42 (48.8%)		Control *n* = 44 (51.2%)	
Female, *n* (%)	67	(77.9)	34	(81.0)	33	(75.0)
Age in years, mean (SD)	50.4	(13.0)	49.5	(13.0)	51.3	(13.1)
BMI kg/m^2^, mean (SD)	34.1	(5.6)	34.1	(6.4)	34.1	(4.8)
≥ 30 kg/m², *n* (%)	67	(77.9)	30	(71.4)	37	(84.0)
Gross family income, *n* (%)						
< 400 000 NOK, *n* (%)	31	(36.5)	14	(34.1)	17	(38.6)
≥ 400 000 NOK, *n* (%)	54	(63.5)	27	(65.9)	27	(61.4)
Self-rated health (1–5)^*^,^†^:						
Bad (<3), *n* (%)	24	(28.2)	15	(35.7)	9	(20.9)
Neither good nor bad (=3), *n* (%)	25	(29.4)	11	(26.2)	14	(32.6)
Good (>3), *n* (%)	36	(42.4)	16	(43.4)	21	(46.5)
Education^*^:						
High: University college and/or university, *n* (%)	35	(41.2)	20	(47.6)	15	(34.9)
Middle: Upper-secondary school, *n* (%)	19	(22.4)	13	(31.0)	6	(14.0)
Low: Upper-secondary school or below, *n* (%)	31	(36.5)	9	(21.4)	22	(51.2)
Self-reported reason for HLC attendance:						
Overweight, *n* (%)	74	(86.0)	35	(83.3)	39	(88.6)
Physical activity, *n* (%)	74	(86.0)	38	(81.8)	36	(90.5)
Healthier diet, *n* (%)	72	(83.7)	36	(85.7)	36	(81.8)
Cardiovascular disease, *n* (%)	7	(8.1)	1	(2.4)	6	(13.6)
Musculoskeletal challenges, *n* (%)	35	(40.7)	21	(50.0)	14	(31.8)
Mental challenges, *n* (%)	22	(25.6)	12	(28.6)	10	(22.7)
Quality of motivation:						
Autonomous regulation (1–7), mean (SD)	6.06	(0.9)	6.06	(1.01)	6.03	(0.80)
Controlled regulation (1–7), mean (SD)	4.01	(1.2)	3.93	(1.27)	4.09	(1.13)
Vitality (1–7), mean (SD)^*^	3.90	(1.5)	3.86	(1.41)	3.93	(1.56)
Eating behaviour^*^,^‡^:						
Emotional eating (1–4), mean (SD)	2.38	(0.8)	2.35	(0.83)	2.41	(0.80)
Cognitive restraint (1–4), mean (SD)	2.48	(0.6)	2.44	(0.62)	2.52	(0.67)
Uncontrolled eating (1–4), mean (SD)	2.26	(0.6)	2.20	(0.52)	2.32	(0.68)
Impact of weight on quality of life^*^,^§^:						
Impaired physical functioning (1–5), mean (SD)^‖^	1.95	(1.0)	1.96	(1.0)	1.94	(1.0)
Impaired self-esteem (1–5), mean (SD)^¶^	2.95	(1.2)	2.96	(1.2)	2.94	(1.1)
Life satisfaction (0–10), mean (SD)^**^	5.77	(2.1)	5.45	(2.3)	6.07	(2.0)
Healthy eating, Log mean (SD)^††^	0.77	(0.37)	0.70	(0.39)	0.83	(0.35)
Unhealthy eating, Log mean (SD)^‡‡^	0.10	(0.28)	0.13	(0.28)	0.07	(0.28)

*Abbreviations and symbols:* RCT: randomised controlled trial; SD: standard deviation; BMI: body mass index; NOK: Norwegian Kroner.

* *n* 85.

† Self-rated health (1–5) divided into bad + quite bad/neither good nor bad/good + very good.

‡ Three scales in The Three Factor Eating Questionnaire R18: Higher scores indicate more restraint, uncontrolled, or emotional eating.

§ Two of five domains Impact of weight on quality of life: Higher scores indicate impairment.

‖ Four out of seven items of weight’s impact on self-esteem.

¶ Nine of eleven items of weight’s impact on physical functioning.

** Life satisfaction measured by Cantril’s ladder (0–10): Highest scores indicate best possible life.

†† Healthy eating: frequency of eating fruit, berries and vegetables.

‡‡ Unhealthy eating: frequency of eating of cakes, biscuits, buns and fast food.

Self-rated health (SRH) is an accurate self-perception of an individual’s overall health status, measured by a single item question (“How is your overall health at the moment?”), and has been validated in a Norwegian study (Vie *et al*., 2014). Alternative answers were bad/quite bad/neither good nor bad/good health/very good health. The two most extreme alternatives on both sides of this continuum were combined yielding a three-level variable.

The participants also reported their reasons for attending the HLCs. The alternatives were PA, healthier diet, overweight, musculo-skeletal challenges, mental challenges, diabetes, high blood pressure, cardiovascular disease, lung disease, tobacco cessation, pressure from others (such as GP, family, friends, and employer), or other reasons.

The Treatment Self-Regulation Questionnaire (TSRQ), developed by Levesque et al, assesses motivation for behaviour change or for non-engagement (Likert scale 1–7) (Levesque *et al*., 2007). The scale identifies three types of motivational regulation: autonomous motivation (six items), controlled motivation (six items), and amotivation (not motivated for change) (three items). Autonomous motivation manifests the volition and the interest of the individual, whereas controlled motivation originates either from external pressure or bad conscience. The TSRQ has been validated in several studies, including a Norwegian study (Mildestvedt *et al*., 2007).

Vitality is associated with fulfilment of the three basic psychological needs defined by self-determination theory (SDT) (Ryan *et al.*, 2008). Subjective vitality was measured by three of the seven items from the Subjective Vitality Scale (Likert 1–7), previously used in a Norwegian survey (response categories “strongly disagree” to strongly agree”) (Solberg *et al*., 2012).

Eating behaviour is multifaceted and subject to a wide range of explanatory perspectives (Buchanan and Sheffield, 2017). To assess if psychological aspects of emotional eating affected eating behaviour, the survey included the Three Factor Eating Questionnaire-R18 (TFEQ-R18). The 18 items cover: (1) emotional eating (the tendency to overeat in reaction to negative emotions) (three items); (2) cognitive restraint (the tendency to restrict one’s food intake instead of using physiological cues, hunger, or satiety as regulators of food intake) (six items); and (3) uncontrolled eating (the tendency to overeat and to lose control over one’s eating (nine items)). The scales range from 1 to 4, and higher scores indicate more restraint, uncontrolled, or emotional eating. TFEQ-R18 has been tested and validated in studies of adults in different weight categories in Scandinavia (Karlsson *et al*., 2000).

The Impact of Weight on Quality of Life-Lite Questionnaire (IWQOL-Lite) is an obesity-specific quality of life measure (Kolotkin *et al.*, 2001). The 31 items version covers five domains: physical functioning, SE, sexual life, public distress and work, and have been tested on Norwegian obese adults (Flølo *et al*., 2014). Higher scores indicate the negative impact of weight on quality of life (Likert scale 1–5). Two domains most relevant for this study included six of the 11 items that cover physical function (Cronbach’s Alpha 0.91); and four of the seven items covering SE (Cronbach’s Alpha 0.92). Participants’ life satisfaction was measured using Cantril´s ladder (0–10) (Steptoe *et al.*, 2015). Here, worst possible life equals value zero, and the top value represents best possible life.

### Sample size and statistical power

All the statistical analyses were conducted using IBM SPSS, 25^th^ version. Sample size calculation was based on an estimated 50% improvement in PA (moderate to vigorous intensity defined as >3 metabolic equivalents, METs) in the intervention group, with a power of 80%. Although it is of contested value (Lydersen, 2015), a post-hoc power calculation of this study revealed that we were able to rule out a 10% between-group post-intervention difference in healthy and unhealthy eating between the intervention and the control group.

### Statistical analysis

We reported the means and standard deviations (SDs) and used linear regression analyses to examine if the intervention had an impact on healthy or unhealthy eating 6 months after baseline. First, we examined if the intervention group improved healthy eating or restricted unhealthy eating. Second, we performed this analysis with participants attending the healthy eating intervention as a separate group (Ia), and those who abstained (Ib), and compared both groups with the control group.

Performing the predictor analyses, we merged responses from the 86 completers (intervention and control groups). The results for each predictor were reported by standardised regression coefficients (*b*) with *P*-values from the F-test. The predictors were BMI, SRH, gross family income, education level, reasons for HLC attendance, quality of motivation, vitality, eating behaviour, impact of weight on quality of life, and life satisfaction. We adjusted for gender, age, and randomisation group and compared the potential predictors of healthy or unhealthy eating after 6 months (temporal causal analyses) (Table 2). Identical analyses were performed that, in addition, adjusted for the baseline values of healthy or unhealthy eating (residual change analyses) (Table 3). Interaction analyses were performed evaluating if initial levels of (un)healthy eating modified the effect of the intervention. A *P*-value of <0.05 was accepted as significant.

**Table 2.** Result from unadjusted and adjusted linear regression analyses of healthy eating 6 months after baseline for 86 completers in the Norwegian healthy life centre study, recruited from June 2014 to September 2015 (by predictors at baseline)

Response variables	Healthy eating^‡^ (Temporal causal analyses)			Healthy eating^§^ (Residual change analysis)		
	*b*	95% CI	*P*-value	*b*	95% CI	*P*-value
Adjusting variables						
Female gender^†^	0.30	(0.10, 0.51)	0.004	0.30	(0.10, 0.51)	0.004
Age^†^	0.83	(−0.12, 0.25)	0.449	0.83	(−0.12, 0.25)	0.449
Intervention group^†^	0.06	(−0.15, 0.28)	0.557	0.06	(−0.15, 0.28)	0.557
Healthy eating at baseline^†^				0.49	(0.30, 0.68)	<0.001
Predictors						
BMI kg/m^2^	−0.13	(−0.38, 0.11)	0.267	−0.09	(−0.31, 0.13)	0.385
Gross family income (1–7)^*^	0.19	(−0.001, 0.40)	0.071	0.14	(−0.05, 0.33)	0.140
Self-rated health (1–5)^‖,^^*^						
Good (>3)	0.00	(reference)		0.00	(reference)	
Neither good nor bad (=3)	0.01	(−0.20, 0.19)	0.957	0.02	(−0.17, 0.22)	0.805
Bad (<3)	−0.40	(−0.62, −0.20)	<0.001	−0.29	(−0.50, −0.08)	0.007
Reasons for HLC attendance						
Cardiovascular disease	−0.03	(−0.25, 0.19)	0.777	−0.01	(−0.21, 0.20)	0.938
Musculo-skeletal challenges	−0.24	(−0.46, −0.03)	0.027	−0.18	(−0.38, 0.01)	0.060
Mental problems	−0.10	(−0.31, 0.10)	0.324	−0.08	(−0.27, 0.10)	0.382
Quality of motivation						
Autonomous regulation (1–7)	−0.12	(−0.34, 0.09)	0.265	−0.12	(−0.31, 0.07)	0.213
Controlled regulation (1–7)	0.09	(−0.14, 0.33)	0.429	0.08	(−0.12. 0.28)	0.415
Vitality (1–7)^*^	0.25	(0.03, 0.46)	0.025	0.14	(−0.07, 0.33)	0.190
Eating behaviour (1–4)^¶,^^*^						
Emotional eating	−0.01	(−0.24, 0.19)	0.808	−0.08	(−0.27, 0.12)	0.436
Cognitive restrain	0.19	(−0.02, 0.40)	0.072	0.10	(−0.09, 0.30)	0.294
Uncontrolled eating	0.17	(−0.05, 0.38)	0.124	0.13	(−0.06, 0.32)	0.178
Impact of weight on quality of life^**,^^*^						
Impaired physical functioning^††^	−0.07	(−0.28, 0.14)	0.506	−0.06	(−0.25, 0.13)	0.549
Impaired Self-esteem^‡‡^	−0.29	(−0.53, −0.05)	0.019	−0.17	(−0.39, 0.06)	0.149
Life satisfaction (0–10)^§§,^^*^	0.24	(0.04, 0.45)	0.002	0.16	(−0.04, 0.34)	0.107

*Abbreviations and symbols*: RCT: randomised controlled trial; SD: standard deviation; CI: confidence interval; HLC: healthy life centre; BMI: body mass index; *b:* standardized regression coefficient (beta).

* *n* 85.

† Unadjusted models.

‡ Temporal causal analyses: models adjusted for gender, age, and group allocation.

§ Residual change analysis: models adjusted for gender, age, group allocation, and Healthy eating at baseline.

‖ Self-rated health (1–5) divided into bad + quite bad/neither good nor bad/good + very good.

¶ The Three Factor Eating Questionnaire: Higher scores indicate more restraint, uncontrolled, or emotional eating.

** Two of five domains in Impact of weight on quality of life-lite: Higher scores indicate greater impact of weight.

†† Four out of seven items of weight’s impact on self-esteem.

‡‡ Nine of eleven items of weight’s impact on physical functioning.

§§ Life satisfaction measured by Cantril’s ladder (0–10): Highest score indicate best possible life.

**Table 3.** Result from unadjusted and adjusted linear regression analyses of unhealthy eating 6 months after baseline for 86 completers in the Norwegian healthy life centre study, recruited from June 2014 to September 2015 (by predictors at baseline)

Response variables	Unhealthy eating^‡^ (Temporal causal analyses)			Unhealthy eating^§^ (Residual change analysis)		
	*b*	95 % CI	*P*-value	*b*	95 % CI	*P*-value
Adjusting variables:						
Female gender^†^	−0.16	(−0.37, 0.05)	0.142	−0.16	(−0.37, 0.05)	0.142
Age^†^	−0.06	(−0.37, 0.19)	0.570	−0.06	(−0.37, 0.19)	0.570
Intervention group^†^	0.18	(−0.03, 0.40)	0.085	0.18	(−0.03, 0.40)	0.085
Unhealthy eating at baseline^†^				0.31	(0.10, 0.17)	0.004
Predictors:						
BMI kg/m^2^	0.36	(0.14, 0.59)	0.001	0.32	(0.10, 0.54)	0.004
Gross family income (1–7)^*^	0.33	(0.12, 0.53)	0.002	0.28	(0.08, 0.49)	0.007
Self-rated health (1–5)^‖,^^*^						
Good (>3)	0.00	(reference)		0.00	(reference)	
Neither good nor bad (=3)	−0.01	(−0.25, 0.24)	0.964	−0.12	(−0.23, 0.20)	0.917
Bad (<3)	−0.07	(−0.31, 0.17)	0.566	−0.03	(−0.25, 0.20)	0.824
Reasons for HLC attendance:						
Cardiovascular disease	−0.18	(−0.40, 0.04)	0.108	−0.20	(−0.42, 0.02)	0.071
Musculo-skeletal challenges	−0.25	(−0.47, −0.03)	0.024	−0.21	(−0.43, 0.00)	0.052
Mental problems	−0.15	(−0.36, 0.06)	0.162	−0.18	(−0.38, 0.02)	0.083
Quality of motivation:						
Autonomous regulation (1–7)	−0.20	(−0.41, 0.02)	0.074	−0.18	(−0.39, 0.03)	0.085
Controlled regulation (1–7)	−0.09	(−0.33, 0.14)	0.425	−0.10	(−0.32, 0.13)	0.394
Vitality (1–7)^*^	−0.04	(−0.26, 0.18)	0.726	−0.03	(−0.23, 0.18)	0.793
Eating behaviour^¶,^^*^:						
Emotional eating (1–4)	0.14	(−0.07, 0.35)	0.196	0.12	(−0.08, 0.33)	0.249
Cognitive restraint (1–4)	−0.22	(−0.43, −0.01)	0.044	−0.16	(−0.37, 0.05)	0.138
Uncontrolled eating^‡^	0.19	(−0.02, 0.40)	0.080	0.14	(−0.07, 0.35)	0.196
Impact of weight on quality of life^**,^^*^:						
Impaired physical functioning (1–7)^††^	0.26	(0.05, 0.47)	0.016	0.22	(0.01, 0.42)	0.038
Impaired self-esteem (1–7)^‡‡^	0.04	(−0.21, 0.29)	0.761	0.07	(−0.18, 0.32)	0.583
Life satisfaction (0–10)^§§^	0.05	(−0.18, 0.27)	0.672	0.05	(−0.17, 0.05)	0.647

*Abbreviations and symbols*: RCT: Randomised controlled trial; SD: standard deviation; CI: confidence interval; HLC: healthy life centre; BMI: body mass index; *b*: standardized regression coefficient (beta).

* *n* 85.

† Unadjusted models.

‡ Models adjusted for gender, age, and group allocation.

§ Models adjusted for gender, age, group allocation, Unhealthy eating at baseline.

‖ Self-rated health (1–5) divided into bad + quite bad/neither good nor bad/good + very good.

¶ The Three Factor Eating Questionnaire: Higher scores indicate more restraint, uncontrolled, or emotional eating.

** Two of five domains in Impact of weight on quality of life-lite: Higher scores indicate greater impact of weight.

†† Four out of seven items of weight’s impact on self-esteem.

‡‡ Nine of eleven items of weight’s impact on physical functioning.

§§ Life satisfaction measured by Cantril’s ladder (0–10): Highest score indicate best possible life.

## Results

The baseline characteristics of the 118 participants and characteristics of dropouts in the study are reported in greater detail elsewhere (Samdal *et al*., 2018a; Samdal *et al*., 2018b; Samdal, 2021).

Six months after baseline, 42 participants in the intervention group and 44 in the control group remained in the study (Table 1). Most of the completers were obese (78% with BMI > 30), female (78%), with bad SRH (58%). Thirty-seven per cent reported a low education level, and 41% had a high education level. The most frequent reasons for attending the HLCs were overweight, PA, healthier diet or musculo-skeletal challenges, and their motivation for behaviour change was highly autonomous. The most common eating behaviour was cognitive restraint, and quality of life was negatively impacted, particularly because of impaired weight-related SE.

The standard period for the HLC interventions could be extended after 3 months, and the average attendance was, in fact, 6 months (data not shown). During this period, the intervention group received a PA intervention, and 27 of these also attended the Healthy Eating intervention (Table 4). We revealed no difference in healthy or unhealthy eating in the intervention group, compared with the controls. However, there was a significant intervention effect in healthy eating for those who received the Healthy Eating intervention (Ia), compared with the control group. This intervention did not reduce unhealthy eating compared with controls.

**Table 4.** Results from multiple regression analyses of change in healthy and unhealthy eating 6 months from baseline for 86 completers in the Norwegian healthy life centre study (RCT), recruited from June 2014 to September 2015

Outcome group	Baseline			Post intervention			Regression		
	*n*	Mean	SD	*n*	Mean	SD	*b*	95% CI	*P*-values
Healthy eating^*^									
HLC interventions	42	0.70	0.39	42	0.86	0.28	0.16	(−0.03, 0.35)	0.108
Control group	44	0.83	0.35	44	0.83	0.28		(reference)	
Ia: HLC interventions, including Healthy eating education	27	0.69	0.37	27	0.92	0.28	0.24	(0.04, 0.43)	0.019
1b: HLC interventions (no Healthy eating education)	15	0.71	0.43	15	0.77	0.27	-0.02	(−0.20, 0.17)	0.860
Unhealthy eating^†^									
HLC interventions	42	0.13	0.28	42	0.15	0.31	0.18	(−0.04, 0.39)	0.100
Control	44	0.07	0.28	44	0.04	0.25		(reference)	
Ia: HLC interventions, including Healthy eating education	27	0.16	0.29	27	0.15	0.31	0.17	(−0.06, 0.39)	0.142
1b: HLC interventions (no Healthy eating education)	15	0.08	0.27	15	0.14	0.33	0.13	(−0.09, 0.35)	0.253

*Abbreviations and symbols*: RCT: randomised controlled trial; SD: standard deviation; CI: confidence interval; HLC: healthy life centre; *b* standardised regression coefficient (beta) adjusted for baseline value of randomisation and healthy or unhealthy eating.

* Healthy eating: frequency of eating fruit, berries and vegetables.

† Unhealthy eating: frequency of eating of cakes, biscuits, buns and fast food.

After performing regression analyses on pooled data for each of the adjusting variables, we entered these variables in models entering the predictor variables one by one in adjusted analyses (temporal causal analyses) (Table 2). Healthy eating at 6 months was impacted by feeling vital and satisfied with one’s life, and not having musculo-skeletal challenges, bad health, and impaired SE. When we also adjusted for healthy eating at baseline (residual change analysis), none of the variables predicted change in healthy eating, except good SRH compared with bad SRH.

In temporal causal analyses of unhealthy eating at follow-up (adjusting for gender, age, group allocation), the strongest predictors were high BMI and impaired physical functioning, together with a high income (Table 3). Unhealthy eating was also predicted by the tendency not to restrain one’s intake of food and by not having musculo-skeletal challenges. When we adjusted for the level of unhealthy eating at baseline (residual change analyses), change in unhealthy eating was still predicted by high BMI and impaired physical functioning, together with a high income. Other individual characteristics could not explain change in healthy or unhealthy eating. Neither autonomous motivation, intention to learn about a healthier diet, or education level could explain healthy or unhealthy eating at follow-up. We could not reveal any interactions between intervention and initial levels of (un)healthy eating (data not shown).

## Discussion

### Main results

This RCT showed that the HLC interventions had no effect on healthy and unhealthy eating 6 months after baseline, compared with controls. However, there was a statistically significant and modest improvement in healthy eating for the subgroup who was offered and accepted the additional healthy eating education, compared to the controls. There was no effect on unhealthy eating for the same subgroup, compared with controls.

### Predictors of healthy and unhealthy eating

SRH, vitality, SE, life satisfaction, and the absence of musculo-skeletal challenges significantly predicted eating more fruit, berries, and vegetables at follow-up. The impact of these predictors on the *change* in healthy eating during the 6 months was, however, insignificant, except for SRH.

High BMI and family income and impaired weight-related physical functioning significantly predicted eating more unhealthily at follow-up, whereas musculo-skeletal challenges and cognitive restraint predicted eating less unhealthily. The residual change analyses confirmed that high income, BMI, and impaired physical function explained an increase in unhealthy eating (residual change) during the 6 months.

### Our findings compared with other studies

#### Healthy eating

There is substantial variability in how intervention studies measure dietary improvements, e.g. reduction in energy intake (kcal/day) or dietary fat (%), increase in fruit and vegetable consumption, or in vegetable or fruit consumption per week (Denison *et al*., 2014; Samdal *et al*., 2017; O’Connor *et al*., 2020). The differences in outcome measures make it difficult to compare the effect across studies, and to understand the interventions’ clinical importance for health improvement. However, a systematic review of 26 RCTs of behaviour change interventions to improve diet found a modest effect at short term (≤ 6 months) with a declining effect over time (≥12 months). The participants resembled the participants in the HLCs’ intervention (mean age ≥ 40, mean BMI ≥ 30, with risk of NCDs).

Three RCTs identified a positive effect of behaviour change interventions (≥ 12 weeks duration, targeting two behaviours: PA and diet (fruit and vegetable consumption) (Annesi, 2013; Gray *et al*., 2013; Patrick *et al*., 2011). Annesi and colleagues suggested that interventions that focus on change in both activity and diet have a reciprocal relationship, where each behaviour reinforces the other (Annesi and Porter, 2013). This was supported in a 1-year RCT of weight management in women. The rationale for this is the robust relationship between exercise and mood improvement, where PA might reduce emotional eating (most often unhealthy food), while a better diet may also affect mood and improve self-regulation of PA (Mata *et al*., 2009).

In the present study, healthy eating was explained by several characteristics of physical and mental health that are the result of optimal regulation of eating behaviour (Verstuyf *et al*., 2012). According to SDT, healthy eating regulation will depend on satisfaction of three basic psychological needs: autonomy, competence, and relatedness, and this can explain how good SRH, high vitality, and life satisfaction impacted participants to eat more healthily.

RCTs of similar interventions (≥12 weeks, change of two behaviours) and similar participants (mean age ≥40, mean BMI ≥ 30, with risk of NCDs) showed no effect on fruit and vegetable consumption (Hardcastle *et al.*, 2008) or just fruit consumption (Morgan *et al*., 2011). These results were explained by a much greater initial concern for inactivity than for an unhealthy diet among the participants (Hardcastle *et al*., 2008). This could also be the case for the HLCs, as increasing PA constitutes their main activity and core competence. Already eating healthily at baseline explained most of the effect on healthy eating at follow-up, and when we adjusted for this in the analyses (residual change analysis), the effect of other predictors was attenuated, and most of them became statistically insignificant. The only exception was bad SRH, predicting a decrease in healthy eating.

#### Unhealthy eating

Two RCTs have identified reduction in unhealthy food, e.g. dietary fat (%) (Assuncao *et al*., 2010; Anderson *et al.*, 2014) and energy intake (kcal/day) (Befort *et al.*, 2008). In the present study, the additional healthy eating intervention made the participants eat more healthy food, but they did not reduce consumption of energy-dense food, for example the intake of cake, biscuits, buns, and fast food, compared with controls. Unhealthy eating at follow-up was predicted by high BMI, high family income, reduced physical functioning, the presence of musculo-skeletal challenges, and the tendency *not* to restrain food intake. Only high family income and impaired physical functioning remained statistically significant in the residual change analysis.

### Strengths and limitations

The strength of this study is the pragmatic randomised controlled design in a primary health care setting, involving both rural and urban municipalities. Randomisation procedures assured random sequence generation and allocation concealment. The intervention group was not blinded, but we used an identical, self-administered questionnaire at the different time-points, introducing a common method bias. Selection bias represents a threat to external validity. The main reason for refusing to participate was having to wait 6 months for the interventions if randomised to the control group. Being asked about health behaviour may produce changes in behaviour, especially in already motivated participants. An attrition rate of 30% affected the statistical power of the study, but the dropouts were equally distributed in both groups. Adaption of the HLC model to local context limits our ability to identify elements in the interventions that were effective. PA interventions were offered at all HLCs, but fewer than two out of three participants in the intervention group received the dietary intervention.

The statistical power analysis was based on the PA outcome. Therefore, the lack of between group differences in change of healthy and unhealthy eating may be due to type II error. The post hoc power analysis showed, however, that only differences in diet scores less than 10% were apt to erroneous omission.

## Conclusion

The study revealed a modest effect on healthy eating limited to participants receiving the healthier eating education, compared to controls. There was no effect on unhealthy eating for the same subgroup. No effects of educational differences were revealed and contrary to common beliefs, higher income predicted unhealthier eating as time passed. It is essential to develop methods and techniques in counselling that work for those most in need. The study suggests that the effect of primary health care interventions to change diet behavior may be improved by an emphasis on developing the participant’s positive self-concepts like SRH, vitality, life satisfaction, and SE.

## Implications

Former HLC studies indicate that PA is not maintained long term, whereas quality of life is increased and sustained (Samdal *et al*., 2018a; Blom *et al*., 2020). The HLC service focused on participants at risk for or who already have developed NCDs. The service must be complemented by population tailored measures in order to promote healthier lifestyle in the society as a whole.
